# CD40 Expression in Fibrocytes Is Induced by TSH: Potential Synergistic Immune Activation

**DOI:** 10.1371/journal.pone.0162994

**Published:** 2016-09-15

**Authors:** Tünde Mester, Nupur Raychaudhuri, Erin F. Gillespie, Hong Chen, Terry J. Smith, Raymond S. Douglas

**Affiliations:** 1 Department of Ophthalmology and Visual Sciences, University of Michigan Medical School, Ann Arbor, Michigan, 48105, United States of America; 2 Department of Ophthalmology of Union Hospital, Medical College, Huazhong University of Science and Technology, Wuhan, Hubei, 430022, People's Republic of China; 3 Internal Medicine, University of Michigan Medical School, Ann Arbor, Michigan, 48105, United States of America; 4 Ann Arbor Veterans Administration Medical Center, Ann Arbor, Michigan, 48105, United States of America; Baylor College of Medicine, UNITED STATES

## Abstract

**Context:**

Fibrocytes appear to participate in inflammation and tissue remodeling in patients with thyroid-associated ophthalmopathy (TAO). These patients have increased frequencies of circulating TSH receptor (TSHR)- and CD40-positive fibrocytes, suggesting TSHR and CD40 may play roles in proinflammatory cytokine production, which ultimately leads to orbital inflammation and tissue remodeling.

**Objective:**

To investigate the potential interactions between the TSHR and CD40 signaling pathways and their roles in IL-6 and TNF-α production.

**Design and Outcome Measures:**

CD40 expression on fibrocytes was assessed using flow cytometry; IL-6 and TNF-α protein release using Luminex technology; increased IL-6 and TNF-α mRNA abundance, using real-time PCR; TSH- and CD40 ligand (CD40L)-stimulated Akt phosphorylation in fibrocytes, by western blot analysis; TSHR-CD40 protein-protein interaction, using co-immunoprecipitation, and CD40-TSHR co-localization, using immunocytochemistry.

**Results:**

TSH enhances CD40 expression at a pre-translational level in fibrocytes. Production of IL-6 and TNF-α after costimulation with TSH and CD40L was greater than that after TSH or CD40L stimulation alone. TSH and CD40L costimulation also resulted in greater Akt phosphorylation. Akt and nuclear factor (NF)-κB inhibitors significantly reduced cytokine production after TSH and CD40L costimulation. TSHR and CD40L are colocalized on the cell surface and form a complex.

**Conclusions:**

TSHR and CD40 in fibrocytes appear to be physically and functionally related. TSH stimulates CD40 production on the fibrocyte surface. Cytokine expression upon simultaneous stimulation of TSHR and CD40 is greater than levels achieved with TSH or CD40L alone. Increased expression of CD40 by TSH is a potential mechanism for this process.

## Introduction

Graves’ disease (GD) is typically characterized by circulating autoantibodies, thyrotoxicosis, and often, orbital inflammation, and orbit remodeling. Antibodies to the TSH receptor (TSHR) stimulate thyroid hormone production by activating this receptor on thyroid epithelial cells resulting in hyperthyroidism.[1] Thus TSHR activation is a central component of GD. Several studies have implicated other proteins, including insulin-like growth factor 1 receptor (IGF-1R), CD40, and major histocompatibility class II molecules, as autoantigens or immune modulators that promote various manifestations of the disease.[2, 3]

Thyroid-associated ophthalmopathy (TAO) is characterized by inflammation of orbital connective tissue and fat. A growing body of evidence indicates that orbital fibroblasts (OFs) play an important role in the pathology of the disease by interacting with infiltrating immune cells, such as T and B cells. [4] Orbital fibroblasts from Graves patients produce hyaluronan and proinflammatory cytokines.[5, 6] One potential mechanism of T cell—OF interaction involves the interaction between CD40 and CD40L. Orbital fibroblasts from patients with TAO expresses CD40 while fibroblasts from patients with non-inflammatory conditions do not.[7] Increased CD40L expression has been observed in T cells derived from the plasma of patients with GD.[8] The CD40-CD40L interaction promotes the production of proinflammatory cytokines, including IL-8 and IL-6.[7] Orbital fibroblasts from patients with TAO also produce cytokines (e.g. IL-6, and IL-8) in response to TSH stimulation, suggesting that circulating antibodies may directly support orbital inflammation.[9, 10]

Fibrocytes have a role in autoimmune diseases, such as rheumatoid arthritis and pulmonary fibrosis.[11, 12] Fibrocytes from the peripheral blood infiltrate sites of inflammation and mediate immune responses and fibrosis. We have demonstrated that the circulating fibrocyte count is higher in patients with TAO than in healthy controls.[13] Fibrocytes express surface markers characteric of fibroblasts (collagen type 1) and hematopoietic cells (CD34 and CD45). These fibrocytes infiltrate the orbit, such that their derivative fibroblasts from TAO patients display a unique phenotypic composition (CD34^+^ CD45^+^ Col1^+^).[6, 13]

We previously observed an increased frequency of CD40^+^ and TSHR^+^ circulating fibrocytes in persons with GD, compared with healthy individuals.[14, 15] TSH or CD40L stimulation of fibrocytes from healthy controls and patients with Graves’ disease resulted in increased production of an array of proinflammatory cytokines, including, IL-6, IL-8, and TNF-α, indicating a potential role for these signaling pathways in orbital inflammation and remodeling.

We report here that the TSHR and CD40 pathways mutually enhance cytokine production in fibrocytes. TSH stimulates increased expression of CD40 on the surfaces of fibrocytes. CD40 and TSHR also appear to be physically associated. Simultaneous stimulation of TSHR and CD40 is synergistic.

## Materials and Methods

### Materials

Histopaque-1077 and sodium azide were purchased from Sigma-Aldrich (St. Louis, MO); Dulbecco’s minimal Eagle’s medium (DMEM), Dulbecco's phosphate-buffered saline (DPBS), Gibco fetal bovine serum (FBS), and Gibco penicillin-streptomycin mixture (Pen Strep) were purchased from Life Technologies (Grand Island, NY). Bovine TSH and Akt inhibitor IV (AKTi) were purchased from Calbiochem EMD Biosciences (La Jolla, CA). Soluble CD40L (MegaCD40L) was purchased from Enzo Life Sciences (Farmingdale, NY). The nuclear factor (NF)-κB inhibitor MG132 was provided by Cayman Chemical (Ann Arbor, MI).

### Patients’ Blood Samples

Patients with GD (n = 4) were recruited from the Kellogg Eye Center at the University of Michigan. The study was reviewed and approved by the Institutional Review Board of the University of Michigan Health System. Written informed consent was obtained from patients in compliance with policies of the Institutional Review Board of the University of Michigan Health System. Research methods followed the tenets of the Declaration of Helsinki.

### Fibrocyte Isolation

Fibrocytes were generated from peripheral blood mononuclear cells (PBMCs) isolated from leukocyte reduction filters provided by the American Red Cross or from the blood of patients with GD. PBMCs were isolated by using the Histopaque-1077 method described by Bucala et al.[16] Isolated PBMCs were washed with DPBS twice. After washing, the pelleted PBMCs were resuspended in DMEM with 10% FBS for culturing fibrocytes.

### Cell Cultures and Treatments

Fibrocytes were cultivated according to the method described by Douglas et al.[13] Each culture well of a 6-well plate was inoculated with approximately 10^7^ PBMCs in 3 mL DMEM supplemented with 10% FBS and 1% Pen Strep. Cultures were incubated in incubators at 37°C under an atmosphere containing 5% CO2. Nonadherent cells were removed after 7 days of culture, and the medium was changed every 3–4 days after the first week. The purity of the fibrocytes reached approximately 90% after 14 days of culture, as confirmed by flow cytometry using anti CD45, CD34, type-1 collagen antibodies (see details below).

Twenty-four hours before TSH and/or CD40L stimulation, the medium was replaced with DMEM containing 1% FBS. CD40L and TSH were added to cultures at final concentrations of 100 ng/mL and 5 mU/mL, respectively, on the basis of previous dose-response experiments. In some experiments, fibrocytes were pretreated with 100 nM AKTi or 5 μg mL^-1^ MG132, for 1 hour before stimulation.

### RNA Isolation and Quantitative Real-Time-PCR

RNA was isolated by using the Aurum Total RNA Mini Kit from Bio-Rad (Hercules, CA). The QuantiTect Reverse Transcription Kit (Qiagen, Valencia, CA) was used for the subsequent reverse transcriptase reaction. Changes in the relative messenger RNA (mRNA) levels of CD40, IL-6, and TNF-α in cultivated fibrocytes due to stimulation were measured by quantitative real-time PCR using the SYBR Green technique (Bio-Rad) with a Bio-Rad CFX96 thermocycler. The following primers were used: for CD40—forward primer, 5’-AGAGTTCACTGAAACGGAATGCC-3’, and reverse primer, 5’-ACAGGATCCCGAAGATGATGG-3’; for IL-6—forward primer, 5’-TGAGAAAGGAGACATGTAACAAGAGT-3’, and reverse primer, 5’-TTGTTCCTCACTACTCTCAAATCTGT-3’; and for TNF-α—forward primer, 5’-GTCTCCTACCAGACCAAG-3’, and reverse primer, 5’-CAAAGTAGACCTGCCCAGACTC-3’. The glyceraldehyde-3-phosphate dehydrogenase gene, *GAPDH*, was used as the internal control, by using the forward primer, 5’-TTGCCATCAATGACCCCTT-3’, and the reverse primer, 5’-CGCCCCACTTGATTTTGGA-3’.

### Luminex Analysis of Extracellular IL-6 and TNF-α Proteins

The levels of extracellular IL-6 and TNF-α produced by cultivated fibrocytes were determined by analyzing culture media collected after 24 hours of treatment with TSH or CD40L. Culture media were subsequently treated with protease inhibitor (Halt Protease Inhibitor Cocktail, Thermo Scientific, Rockford, IL), aliquoted, and frozen at -80°C until use. Cytokine levels in the media obtained from unstimulated and stimulated cultures were determined by using Luminex technology with IL-6 human and TNF-α singleplex bead kits (Life Technologies, catalogue numbers LHC0061 and LHC3011, respectively).

### CD40 Protein Determination Using Flow Cytometry

After treatment of PBMCs with TSH for 24 h, fibrocytes were analyzed by using flow cytometry. Cells were gently scraped off the bottoms of the wells and collected by centrifugation (500 × *g*, 5 min). Cells were washed with staining buffer (SB) containing DPBS, 2% FBS, and 0.1% sodium azide. The following antihuman, fluorochrome-conjugated antibodies were added: CD40-PE (catalogue no. 555589), mouse IgG1, ĸ isotype control-PE (catalogue no. 554680), and CD34 PE-Cy7 (catalogue no. 560710), all from BD Biosciences (San Jose, CA); CD45-PerCP (catalogue no. MHCD4531) from Life Technologies; and TSHR-PE (catalogue no. 53542) from Santa Cruz Biotechnology (Santa Cruz, CA). Cells were incubated with the antibodies for 30 min at 4°C in the dark and then washed twice with SB.

To stain intracellular collagen I, cells were permeabilized with Cytofix/Cytoperm (BD Biosciences, catalogue no. 554722) for 20 min at 4°C, washed twice, and resuspended in 100 μL Perm/Wash buffer (BD Biosciences, catalogue no. 554723). Collagen type I-FITC antibodies (catalogue no. FCMAB412F) from Millipore (Temecula, CA) were added to the cells and incubated for 30 min in the dark on ice. Cells were washed again, twice, and fixed with 1% paraformaldehyde. Analysis was performed with an LSR II flow-cytometer (BD Biosciences). At least 1 × 10^6^ events were collected for each sample. Mean fluorescence intensity (MFI) was defined as a ratio of the geometric mean fluorescence of the sample to the geometric mean fluorescence of the isotype control, in which a mean fluorescence intensity of 1 indicates that the fluorescence intensity of the sample is the same as that of the background or isotype.

The number of CD40 antibodies bound per cell (ABC) was estimated by using QuantiBRITE PE from BD Biosciences (catalogue no. 340495).

### Co-Immunoprecipitation of CD40 and TSHR, and Western blot

CD40 (41/CD40, BD Transduction Laboratories, catalogue no. 611363) and TSHR (A10) antibodies were purchased from BD Biosciences and Advanced Targeting Systems (catalogue no. AB-N16, San Diego, CA), respectively. Anti pAkt (Ser 473) (catalogue no. 5171), and β-actin antibodies (catalogue no. 3700 were from Cell Signaling (Boston, MA).

The protein-protein interaction between CD40 and TSHR was analyzed by using co-immunoprecipitation (co-IP). Cultured fibrocytes were collected by centrifugation at 4000 × *g* for 10 min and treated with protease inhibitors (Halt Protease Inhibitor Cocktail, Thermo Scientific). The cell pellet was resuspended in 100 μL ice-cold RIPA buffer (Thermo Scientific) and incubated at 4 °C for 10 min. Cell debris was removed by centrifugation (10 000 × *g* for 10 min). The resulting protein extract (approx. 0.5 mg protein) was incubated with 50 μL of protein A agarose beads (SC-2001, Santa Cruz Biotechnology), coupled with anti-CD40 antibody or anti-TSHR antibody, at 4 °C, by using the procedure recommended by Santa Cruz Biotechnology. Immunoprecipitates were resuspended in Laemmli buffer (Bio-Rad) and loaded onto a gradient (4–20%) Tris/Glycine SDS-PAGE gels. Precipitated TSHR and CD40 were visualized by consecutive Western blot analysis by using the aforementioned antibodies.

### Immunofluorescence Microscopy

To study the colocalization of CD40 and TSHR, fibrocytes were cultivated on 8-chamber slides for 12–14 days. Cells were harvested and fixed in 4% paraformaldehyde, then blocked with DPBS containing sheep serum (20%). Anti-TSHR (sc-13936) and -CD40 (sc-65263) antibodies from Santa Cruz Biotechnology were used together with secondary antibodies Alexa Fluor 448 donkey antirabbit IgG (A-21206) and Texas Red goat anti–mouse IgG (T-862) from Life Technologies. Cells were mounted in Vectashield medium (Vector Laboratories, Burlingame, CA) and examined with a confocal microscope (model SP5, Leica Microsystems, Wetzlar, Germany).

### Statistics

Statistical analyses were performed using a two-tailed t-test or a one-way ANOVA with Tukey's multiple comparison posttest, as appropriate. Data are reported as the mean ± standard deviation. All experiments were repeated at least three times.

## Results

### TSH Increases CD40 Expression

**Fig 1:**
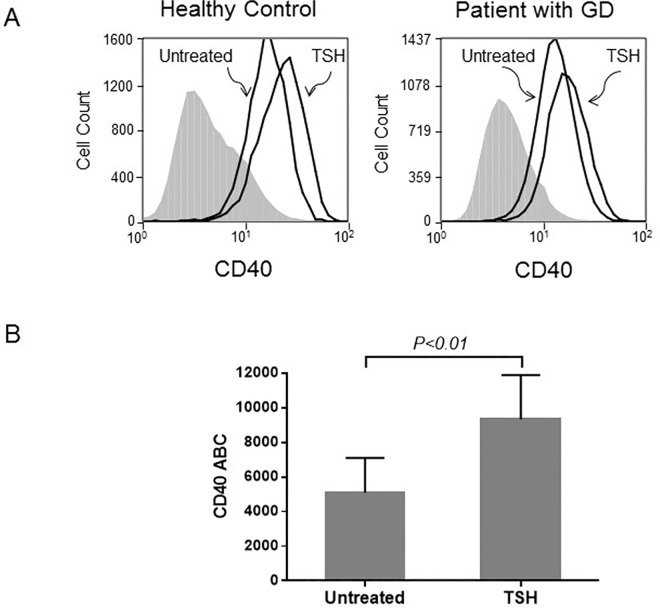
TSH increases the abundance of CD40 protein on the surface of fibrocytes. (A) Results of flow cytometric analyses show that the level of cell-surface CD40 is augmented. As shown in representative histograms, 24 h TSH stimulation resulted in comparable CD40 stimulation in fibrocytes from healthy controls and patients with Graves’ diseases (1.34- and 1.50-fold MFI increase, respectively). in cells. (B) The CD40 antibodies bound per cell (ABC) test also shows a statistically significant (n = 6, *P* < 0.01) increase in CD40 density per cell in samples treated with TSH.

Fibrocytes were identified as cells that are CD45, CD34, and type 1 collagen positive by flow cytometric analysis. According to our previous studies, CD40 and TSHR are constitutively expressed in fibrocytes.[14, 15] In the current study, we observed that fibrocytes from healthy controls and patients with GD stimulated with TSH displayed higher levels of CD40 on their surfaces (Fig 1). TSH stimulation augmented the amount of cell-surface CD40, resulting in an increase in mean fluorescence intensity (MFI) from 1.96 ± 0.68 to 2.97 ± 0.98 (n = 4, *P* < 0.05) in healthy controls and from 1.88±0.63 to 3.23±0.78 in patients with GD (n = 4, *P<*0.05) (Fig 1A). As we have observed previously for other proteins [17, 18] TSHR signaling in fibrocytes from healthy donors and patients with GD displayed similar increases in cell-surface CD40 after TSH stimulation. Therefore, consecutive experiments were performed using fibrocytes from healthy donors. ABC test which determines the number of anti-CD40 antibodies bound on individual cells further confirmed the stimulatory effect of TSH on CD40. The ABC test indicated a statistically significant (n = 6, *P* < 0.01) increase in CD40, from 5091±557 to 9339±1043 antibodies per cell (Fig 1B).

**Fig 2:**
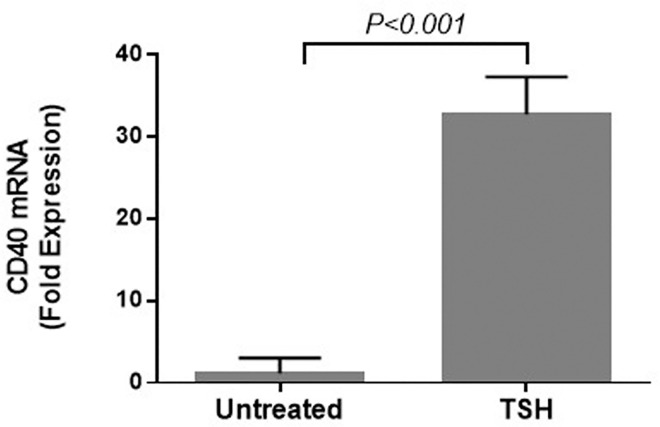
TSH stimulates CD40 expression at the transcriptional level in fibrocytes. The steady-state CD40 transcript level after 6 hours of TSH addition increased 30-fold over baseline.

Because TSH stimulation increased levels of CD40 at the cell surface, we used quantitative real-time PCR to determine mRNA expression. Steady-state CD40 transcript levels showed a 30-fold increase after the addition of TSH (Fig 2). This indicates that TSH stimulates CD40 expression at the pretranslational level and that the increase in CD40 level on the cell surface is not explained solely by migration of existing CD40 to the surface, as was reported previously.[19] We therefore sought to investigate the relationship between these two receptors and their signaling pathways.

### Synergistic Effect of CD40 and TSH Stimulation on Cytokine Production

**Fig 3:**
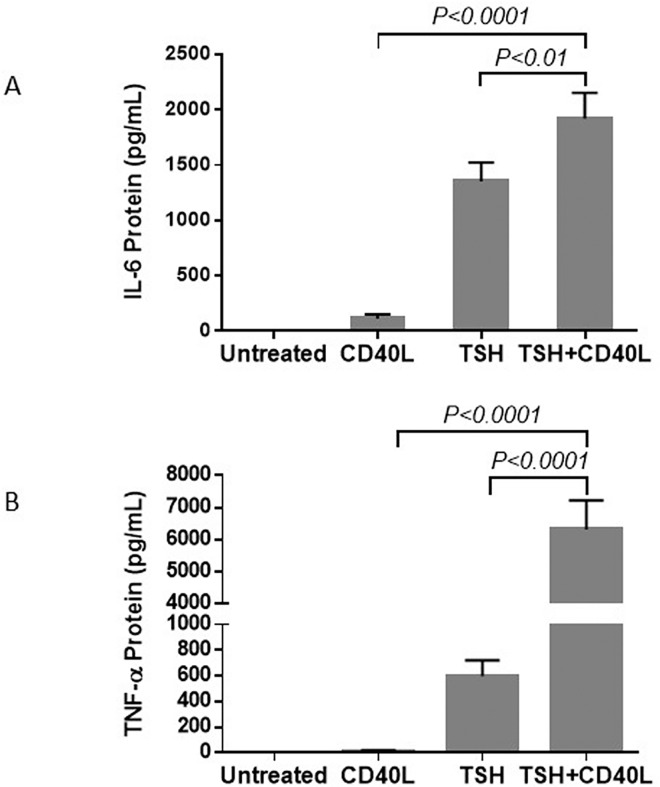
Treatment with a combination of TSH and CD40L stimulates greater cytokine production in fibrocytes than treatment with either CD40L or TSH alone. The titers of IL-6 (A) and TNF-α (B) were measured, using Luminex technology, in culture media collected 24 h after stimulation.

Previous studies showed that stimulation of fibrocytes with TSH or CD40L increases cytokine production.[14, 15] We examined whether the simultaneous addition of TSH and CD40L had any effect on cytokine production. We chose IL-6 and TNF-α as two examples because we had previously confirmed that CD40 and TSH alone consistently stimulate the production of IL-6 and TNF-α by fibrocytes. Luminex technology was used to measure the extracellular titers of IL-6 and TNF-α. When TSHR and CD40 were stimulated simultaneously with their corresponding ligands, greater cytokine production was observed than that observed upon stimulation with CD40L or TSH alone (Fig 3).

**Fig 4:**
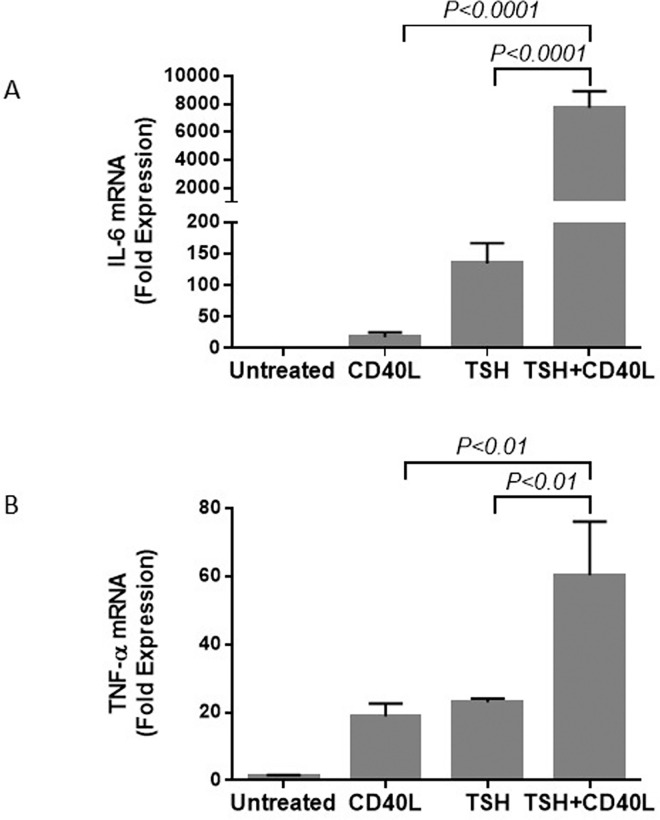
Treatment with a combination of CD40L and TSH increases the levels of IL-6 (A) and TNF-α (B) mRNA more than treatment with either CD40L or TSH alone. Cells were treated with TSH, CD40L, or a combination of these ligands for 6 h.

The combined stimulatory effects on IL-6 and TNF-α production were also observed at the pretranslational level (Fig 4). The trends at the mRNA and protein levels were similar. Stimulation with a combination of TSH and CD40L resulted in a greater increase in IL-6 and TNF-α production than that produced by stimulation with CD40L or TSH alone.

### Signaling Pathways

**Fig 5:**
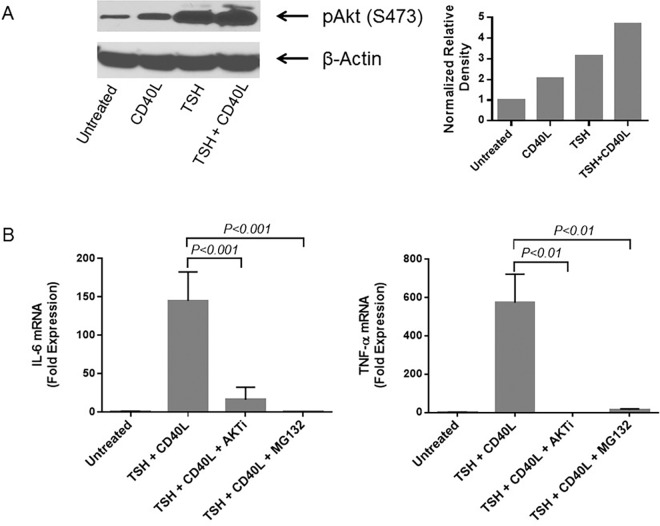
Treatment with a combination of CD40L and TSH increases Akt phosphorylation more than treatment with either CD40L or TSH alone. (A) Western blot analysis used an antibody that recognizes the phosphorylation of S473 in Akt. Densitometric analysis by ImageJ for the results is shown in the right panel. (B) Akt inhibitor IV (AKTi) diminished the stimulatory effect of TSH + CD40L treatment on the production of IL-6 mRNA (*left panel*) and TNF-α mRNA (*right panel*). Similar inhibition of cytokine production was observed when the NF-κB inhibitor, MG132, was added to TSH + CD40L–stimulated fibrocytes, indicating the involvement of NF-κB in TSH and CD40L signaling. AKTi and MG132 were added 1 h before the addition of ligands.

Previously, we showed that Akt is involved in the CD40 signaling that leads to IL-6 and IL-8 production by fibrocytes.[15, 20] We also found that cells stimulated with TSH or CD40L demonstrate rapid Akt phosphorylation at S473.[20] Here, we show that the simultaneous addition of CD40L and TSH further increases Akt phosphorylation, compared with the addition of CD40L or TSH alone (Fig 5A). Densitometry analysis (Fig 5A, right panel) confirm that TSH and CD40L together stimulate Akt S473 phosphorylation greater than TSH or CD40L alone (4.7- vs. 2.1- and 3.1-fold increase, respectively). Adding an AKTi one hour before simultaneous stimulation with TSH and CD40L significantly diminished the increase in IL-6 and TNF-α mRNA production (Fig 5B).

The same inhibition of cytokine production was observed when MG132 was added to TSH + CD40L–stimulated fibrocytes, indicating the involvement of NF-κB in TSH and CD40L signaling (Fig 5B).

### Colocalization of CD40 and TSHR

**Fig 6:**
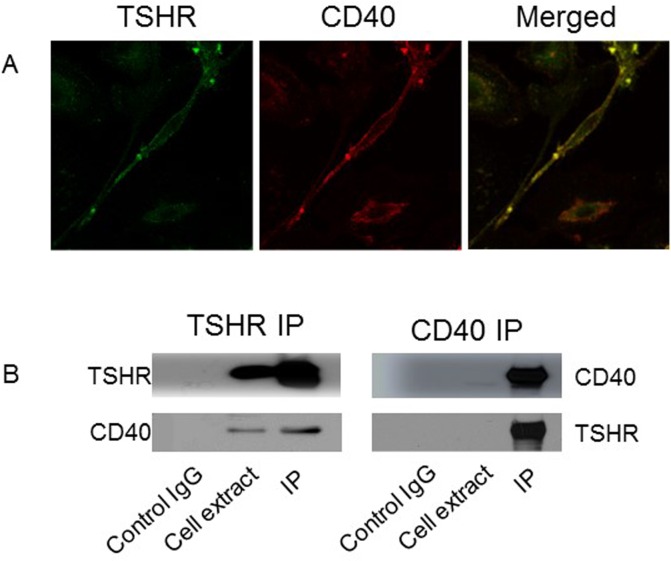
Co-localization and protein-protein interaction of TSHR and CD40 in fibrocytes were confirmed with confocal microscopy and co-immunoprecipitation (co-IP). (A) The spindle-shaped fibrocytes indicate that THSR (green, *left panel*) and CD40 (red, *middle panel*) are colocalized on the cell surface, resulting in the yellow-colored merged image (*right panel*). (B) Co-IP studies using TSHR (*left panel*) or CD40 antibodies (*right panel*) show that TSHR antibody pulls down TSHR and CD40, and CD40 antibody also pulls down both proteins, indicating physical contact between the 2 proteins.

Since TSH stimulates CD40 expression and the simultaneous addition of TSH and CD40L to fibrocyte cultures yields enhanced cytokine production, we sought to determine if these receptors are associated. We found that the TSHR and CD40 receptors colocalize on the cell membrane of fibrocytes using confocal microscopy (Fig 6A). Physical interaction of the receptors was demonstrated by co-immunoprecipitation studies, in which anti-TSHR antibodies immunopreciptate both TSHR and CD40. Anti-CD40 antibody also immunoprecitpates both TSHR and CD40 proteins (Fig 6B).

## Discussion

Fibrocytes are derived from monocytes; they migrate to sites of injury and infiltrate the surrounding tissues, where they promote wound healing and fibrosis.[21, 22] Orbital and thyroid tissues from patients with TAO in contrast to controls exhibit abundant cells phenotypically consistent with fibrocytes.[13] Patients with TAO also have an increased number of circulating fibrocytes[13] expressing both TSHR and CD40.[14, 15] Given the role of fibrocytes in other inflammatory processes, we have hypothesized that fibrocyte infiltration of target tissues and concomitant cytokine production may contribute to the orbital inflammation and tissue remodeling observed in TAO.

Adipocytes, and OFs from patients with GD, express modest levels of TSHR compared to fibrocytes and thyroid tissue.[9, 14, 23] The main physiological function of TSHR appears to be regulation of thyroid hormone production in thyroid epithelial cells. However, its expression by fibrocytes and promotion of cytokine production may suggest a more diverse role in extrathyroidal cells.[6, 24, 25]

In addition to TSHR signaling, the CD40—CD40L interaction has been implicated in GD as it has in other autoimmune diseases.[26] Anti-CD40L therapy has been considered for treatment of autoimmune diseases.[27] A single nucleotide polymorphism in the Kozak sequence of *CD40* is associated with an increased risk for GD.[28] The serum level of soluble CD40L is elevated in active GD.[29] Furthermore, Hwang et. al.[7] recognized that, compared with healthy donors, patients with TAO have not only a greater number of CD40^+^ OFs, but that the surfaces of these CD40+ OFs also have an increased density of CD40. CD40L-expressing T cells have been shown to infiltrate the orbit in patients with TAO[30] and CD40L can also be detected on other cell types, including fibroblasts.[31] These cell types may both contribute to the stimulation of CD40. The CD40-CD40L interaction in orbital fibroblasts promotes the production of hyaluronan[32] and pro-inflammatory cytokines, including IL-8 and IL-6.[7]

We now demonstrate that TSH can increase the expression of CD40 and that the simultaneous stimulation of TSHR and CD40 molecules can mutually enhance cytokine production. The TSH-induced increase in CD40 occurs at the transcriptional level, resulting in a subsequent increase in CD40 protein displayed on the cell surface. The promoter region of the CD40 gene contains several predicted regulatory elements, including four NF-κB binding sites, which are potential candidates for TSH-induced CD40 stimulation. TSHR signaling in fibrocytes follows the Akt—NF-κB pathway; NF-κBp65 relocation to the nucleus was observed.[6] The stabilizing effect of TSH on mRNA may also contribute to the increased CD40 expression.[6, 33]

Orbital tissue from patients with TAO contain significantly more CD40+ cells than that from healthy controls.[7] This observation may be explained by the different cell composition of healthy orbits and TAO orbits, since TAO orbits are infiltrated by CD40 + fibrocytes.[13, 15] Now we can speculate that TSHR signaling in GD can also contribute to increased CD40+ cell population in TAO and thereby further promote inflammation.

We found that TSHR and CD40 co-localize on the fibrocyte surface and co-immunoprecipitation suggests potential protein-protein interaction. TSHR has been shown to physically and functionally interact with other surface receptors. Tsui et al.[34] reported that IGF-1R and TSHR form a physical and functional complex. It is interesting to note that IGF-1R and CD40 do not form an IFG-1R-CD40 complex. We performed pulldown assays with antibodies against CD40 and the α and β subunits of IGF-1R, but could not demonstrate a protein-protein interaction.

The significance of TSHR-CD40 complex formation is not clear. It is also unclear whether the enhanced effect on cytokine production is due solely to the increase in CD40 expression. It is conceivable that the physical interaction between the two receptors enhances each other’s signaling in some other manner. Both signaling pathways involve Akt and NF-κB; co-stimulation has enhanced Akt phosphorylation. Furthermore, the presence of Akt and NF-κB inhibitors reduces the steady-state mRNA levels of IL-6 and TNF-α almost to the levels of untreated cells, as was observed when TSH or CD40L stimulation of cytokines were studied separately.[15, 20] Further interrogation of the function of this complex may provide target(s) for future therapeutics targeting GD and TAO.

In this work, we discovered an alternative function of TSHR signaling, the stimulation of CD40 expression. This observation, along with the discoveries that TSHR forms complexes with CD40, provides additional evidence that TSHR signaling has functional links to other signaling pathways. These findings indicate that THSR may modulate complex cellular events. Further studies may provide insight into these complex interactions.
